# Initial estimates of COVID-19 infections in hospital workers in the United States during the first wave of pandemic

**DOI:** 10.1371/journal.pone.0242589

**Published:** 2020-12-04

**Authors:** Junaid A. Razzak, Junaid A. Bhatti, Muhammad Ramzan Tahir, Omrana Pasha-Razzak

**Affiliations:** 1 Johns Hopkins University School of Medicine, Baltimore, Maryland, United States of America; 2 Manulife Canada, Toronto, ON, Canada; 3 Apotex Inc., Toronto, ON, Canada; 4 Penn State College of Medicine, Hershey, PA, United States of America; University of Botswana Faculty of Medicine, BOTSWANA

## Abstract

**Objective:**

We estimated the number of hospital workers in the United States (US) that might be infected or die during the COVID-19 pandemic based on the data in the early phases of the pandemic.

**Methods:**

We calculated infection and death rates amongst US hospital workers per 100 COVID-19-related deaths in the general population based on observed numbers in Hubei, China, and Italy. We used Monte Carlo simulations to compute point estimates with 95% confidence intervals for hospital worker (HW) infections in the US based on each of these two scenarios. We also assessed the impact of restricting hospital workers aged ≥ 60 years from performing patient care activities on these estimates.

**Results:**

We estimated that about 53,000 hospital workers in the US could get infected, and 1579 could die due to COVID19. The availability of PPE for high-risk workers alone could reduce this number to about 28,000 infections and 850 deaths. Restricting high-risk hospital workers such as those aged ≥ 60 years from direct patient care could reduce counts to 2,000 healthcare worker infections and 60 deaths.

**Conclusion:**

We estimate that US hospital workers will bear a significant burden of illness due to COVID-19. Making PPE available to all hospital workers and reducing the exposure of hospital workers above the age of 60 could mitigate these risks.

## Introduction

The COVID-19 pandemic has resulted in significant loss of life and significant disruption of social and economic structures worldwide [1]. By March 27, 2020, the total number of people infected exceeded 595,000, with over 27,000 deaths [2]. Even robust healthcare systems are challenged severely by the numbers of patients and the severity of the illness [3]. The disease is caused by a novel coronavirus and spreads through respiratory droplets, by direct contact with infected persons, by contact with contaminated objects and surfaces, and through airborne transmission. Disease transmission starts during the incubation period, which lasts for 5–6 days and continues through the initial symptomatic phase of the disease [4].

One of the major concerns during the COVID-19 outbreak has been the safety of healthcare workers, especially those working in hospital settings [5]. By the end of March, Wuhan, China, and Italy had reported over 3,000 and 7000 healthcare worker (HCW) infections, respectively. Italy had also reported 51 deaths amongst physicians [6]. Ongoing shortages of personal protective equipment (PPE) resulted in heightened anxiety and refusal of care by the healthcare providers [7]. In the face of restrictions to PPE availability and usage, and reports of deaths, HCWs remain worried about their health and the health and safety of their families [8]. These concerns are not unfounded as the risk of infection, especially during the initial phase of the pandemic, appeared inordinately high, with 20% of hospital workers in Italy becoming infected [3]. While hospital workers without the requisite PPE continue to see patients in many settings, an increasing reluctance to provide care to patients with COVID-19 remains one of the major risks to the global response in the subsequent surges [5, 9–11].

Despite a greater understanding of disease epidemiology, there is currently no published estimates on the expected number of infected hospital workers [10]. We estimated the number of infections and deaths due to COVID-19 amongst the hospital workers with limited access to PPE and projected the number of lives saved if widespread infection control measures, including PPE, are implemented. We also estimated the morbidity and mortality impact of limiting exposures of hospital workers over 60 years of age.

## Methods

### Study design and settings

Our secondary analyses estimated hospital worker infections in the US based on health worker infections and deaths per 100 deaths from COVID-19 in Hubei and Italy normalized for hospital workers per beds in the US.

### Data sources

We extracted data for COVID-19 infections from Hubei, China, and Italy. We also extracted data from the two other jurisdictions, Wuhan city (located in Hubei province) and South Korea, for initial comparisons. China's Hubei province, the initial site of the epidemic, is a landlocked province with more than fifty-eight million people. Wuhan is a transport hub and major rail interchange in China and reported over 60% of all cases during the first wave of the pandemic in China. Italy, which surpassed China in the number of deaths due to COVID-19 in its first wave [6] with half of the country's cases centered around Bergamo (population 122,000) in Lombardy (population of about 10 million) [12, 13]. South Korea, a nation of 51 million people, is recognized as an exemplar in controlling the spread of the disease and reporting one of the lowest mortality rates in the world [14–16]. We used the publicly available data covering all cases from the beginning of the epidemic till March 19, 2020, for China and South Korea and till March 27 for Italy.

#### Coronavirus COVID-19 global cases by the Center for Systems Science and Engineering (CSSE) at Johns Hopkins University [2]

At the time of the analysis, JHU data was based on several sources. For China, the data was obtained from DXY, an online platform run by members of the Chinese medical community [17]. Every 15 minutes, the cumulative case counts were updated from DXY for all provinces and affected countries and regions in the country. Additionally, for countries and regions outside mainland China (including Hong Kong, Macau, and Taiwan), other sources include Twitter feeds, online news services, and direct communication sent through the regional and local health departments, including the China CDC (CCDC), Hong Kong Department of Health, Macau Government, Taiwan CDC, European CDC (ECDC), the World Health Organization (WHO), were used [2]. For city-level case reports in the U.S., reporting relied on the US CDC. A team at JHU coordinates all manual updates. The data were stratified by country and included total confirmed cases, daily new cases, total confirmed deaths, daily new deaths, and recovered cases [2]. Information was not available on tests or breakdown of confirmed cases.

#### Official government publications

We used the daily report published by the Italian Ministry of Health (English version) to obtain the overall number of COVID-19 infections, deaths amongst those patients, and the number of health worker infections [6]. We also obtained the percentage of patients who were severely or critically ill from the report. For US data, we used the most up to date official press release from the city and state departments of New York [18], Massachusetts [19], and Florida [20].

We used the Organization of Economic Cooperation and Development data for 2017 for numbers of physicians, nurses, and hospital beds in China, Italy, and the United States [21, 22].

The study was exempt from ethics approval as we used only the open-source secondary anonymized data. We followed the STROBE guidelines for reporting.

### Study measures

From all three sites, we extracted data on the total number of COVID-19 infections and deaths in the general population and amongst the healthcare workers. We defined COVID-19 death as those due to suspected, probable, or confirmed COVID-19. Following the definition used by China and Italy, we defined healthcare workers' infection as laboratory-confirmed infection with COVID-19 amongst providers of medical treatment and care services [23].

### Statistical analyses

We estimated the hospital worker infections and deaths in the US in systematic stepwise computations based on observed statistics from Hubei and Italy. Firstly, we computed the COVID-19 deaths per million population. We then calculated the number of admissions based on the proportion of severe and critical cases. Based on reported health worker infections in Hubei and Italy, we estimated the rates of healthcare worker infections per 1,000 admissions and 100 deaths.

We then computed the expected deaths in the US based on COVID-19 deaths per million population in various jurisdictions. We computed the COVID-19-related hospitalizations in the US considering four scenarios: four times the deaths (e.g., Italy), eight times the deaths (e.g., Hubei), ten times the deaths (e.g., close to current NY city), and 15 times the deaths (e.g., similar to Florida). We used then Monte Carlo simulations (1 million iterations) to compute the point estimate with 95% confidence intervals of the number of infections among healthcare workers in the US if the hospitalizations would remain between four and eight times the deaths. These simulations were run for estimates based on Hubei and Italy separately. We adjusted our estimates for the number of hospital workers per bed. There are 5.06 healthcare workers per hospital bed in the US compared to 3.36 in Italy and 1.08 in China [21, 22]. The adjustment was made by a factor of 1.50 for Italian estimates, and 4.67 for Hubei estimates.

We then estimated the number of hospital workers infections if a set of interventions described in prior studies from SARS were implemented. These interventions include availability and use of gowns, gloves, eye protection, and N-95 masks by100% of workers and setting up a triage outside of hospitals (in tents or other shelters); ensuring patients are triaged in outdoor screening stations and delineating contamination, transition, and clean zone, each separated by checkpoints [24, 25]. For estimating infection counts if only the high risk (exposed) workers received PPE, we assumed that 30% of total healthcare would work directly with patients (i.e., high-risk) whereas 70% would be managing other patients as observed in China [26]. For these estimations, we assumed the infection rate would be 55% in high risk (exposed) hospital workers and 26% in other hospital workers under inappropriate PPE conditions [26]. We used the following to compute infections if PPE is available only in high-risk workers.

Infections=(%highriskworkers×infectionrate)+(%lowriskworkers×infectionrate)

Based on the age distribution of infection risks in Italy and China, we also computed COVID-19 infections if hospital workers over the age of 60 or 50 years are restricted from working directly with the patients [6, 27]. For all computations, we estimated the death rate assuming it to be 3% of infections as observed globally for cases up to March 27, 2020 [28].

## Results

As of March 19, 2020, the province of Hubei had 67,800 cases, of which 48,557 were from Wuhan, and South Korea had 8,799 cases. As of March 27, 2020, Italy saw 79,968 infections. The mortality rate per million showed high variability between the regions (Table 1).

**Table 1:** Summary of the total cases and infections and deaths amongst hospital workers in Hubei province, China and Italy.

	S Korea	Hubei	Italy	Wuhan
Population (in millions)	51.47	58.5	60.48	11
Cases (actual)	8799	67800	79968	48557
Deaths (actual)	102	3130	7590	2838
Deaths per million population	1.9	53.5	125.5	258
Hosp admission (10X deaths) per million population	19.8	535	395.2	2580
Hospital workers with COVID-19 infection (actual)		3387	2898	3048
Hospital worker infections per 1000 admissions		163.9	183.7	162.7
Hospital worker infections per 100 deaths		108.2	94.1	107.4

Table 2 shows the projected numbers for death and hospital admission in the US. We used mortality rates from the four regions to estimate US mortality from the highest (Wuhan) to the lowest (South Korea) estimates. We then calculated the expected number of patients based on the admission/death ratio of 4:1, 8:1, 10:1, and 15:1. If the epidemic in the US is as severe as in Wuhan with a similar health system response, then total admission may range from 196,838 to about 738,141 patients. Based on the Hubei scenario, the US could see COVID-19 related hospitalizations from 54,996 to 206,236. Based on the Italian scenario, the COVID-19 admissions in the US could range from 165,379 to 620,170.

**Table 2:** Projected mortality and hospital admissions in the United States given statistics from S. Korea, Hubei, Italy, and Wuhan.

	S Korea scenario	Hubei scenario	Italy scenario	Wuhan scenario
Projected total mortality in the US	652	13749	41355	84998
Admission projections (4X mortality)	2609	54996	165379	196838
Admission projections (8X mortality)	5218	109992	330757	393675
Admission projections (10X mortality)	6523	137490	413447	492094
Admission projections (15X mortality)	9785	206236	620170	738141

Table 3 presents the unadjusted counts for hospital worker infections and deaths for scenarios if admissions were four, eight, ten, or fifteen times the deaths. The highest number assumes a very high-intensity epidemic with many deaths, and the health system is not prepared to handle the surge.

**Table 3:** Unadjusted estimated counts of COVID-19 infections in US hospital workers.

	Hubei Model	Italy Model
Admissions 4X of deaths		
Infections	9017	23588
Admissions 8X of deaths		
Infections	18034	47177
Admissions 10X of deaths		
Infections	22542	58971
Admissions 15X of deaths		
Infections	33814	88456

The Monte Carlo simulations based on Hubei (China) suggested that about 53,640 US hospital workers (95% CI: 43,160 to 62,251) might get infected with COVID-19 after adjusting for differences between US and Chinese workers per beds. Similarly, the Monte Carlo simulations based on Italian estimates suggested that 53,097 US hospital workers (95% CI: 37,133 to 69,003) might get infected with COVID-19 after adjusting for differences between US and Italian workers per beds.

The detailed estimates for hospital worker infections and deaths under different scenarios are presented in Table 4. These estimates suggest that if ideal PPE conditions are implemented only for high-risk healthcare workers, then the US might face 28,100 hospital worker infections (95% CI: 23,048 to 33,242) considering the Hubei (Chinese) scenario and about 28,354 hospital worker infections (95% CI: 19,829 to 36,848) considering the Italian scenario (S1 Fig). Similarly, if hospital workers aged ≥ 60 years are restricted from direct patient care, then the US might face 1,985 hospital worker infections (95% CI: 1,627 to 2,347) based on the Hubei scenario and about 2,002 hospital worker infections (95% CI: 1,400 to 2,602) based on the Italian scenario.

**Table 4:** Adjusted estimated infection and death counts among hospital workers in the US.

	Hubei		Italy	
	n_e_	95% CI	n_e_	95% CI
Infections (Total estimated)	53640	43160–62251	53097	37133–69003
100% PPE for all hospital workers	0*		0*	
100% PPE for high-risk workers	28100	23048–33242	28354	19829–36848
Restricting workers age to < 60y	1985	1627–2347	2002	1400–2602
Restricting workers age to workers < 50y	564	462–667	569	398–739
Deaths (Total estimated)	1579	1295–1868	1592	1114–2070
100% PPE for all hospital workers and patient flow changes	0*		0*	
100% PPE for high-risk workers	843	691–997	851	595–1105
Restricting workers age to < 60y	60	49–70	60	42–78
Restricting workers age to workers < 50y	17	14–20	17	12–22

n_e_ n estimated

95% CI 95% Confidence Intervals

PPE Personal Protective Equipment

* We assume that under ideal PPE conditions, there would be no infections and deaths among hospital care workers

Our analyses showed that death counts in US hospital workers could be 1,579 (95% CI: 1,294 to 1867) based on the Hubei scenario and 1,592 (95% CI: 1,114 to 2,070) based on the Italian scenario. We estimate mortality to be much lower if PPEs were made available to high-risk workers (843; 95% CI: 691 to 997 under the Hubei scenario and 851; 95% CI: 595 to 1105 under the Italian scenario). The restriction of hospital workers aged ≥ 60 years from direct patient care could reduce the death counts to 60 based on the Hubei (95% CI: 49 to 70) and the Italian (95% CI: 42 to 78) scenarios.

## Discussion

We present estimates for the burden of disease and deaths due to COVID-19 amongst hospital workers in the US. We highlight the risks of limited access or use of PPEs amongst hospital workers in the US and estimate the impact of two interventions: infection control, including the use of PPEs and age restriction of hospital-based healthcare workers. We also present a clear path to significantly reducing this burden through two strategies: continuous widespread and proper use of personal protection strategies and limiting the exposure to hospital workers over the age of 60.

Unlike the community's risk of exposure, we based the risk of hospital worker infections on the magnitude of exposure defined as the number of patients with COVID-19 admitted to the hospitals across the US. We believe that hospital admissions and deaths, rather than the total number of cases in the community, are more useful measures of the risks to hospital workers and are critical metrics of the burden on the healthcare system.

Our study demonstrates the potential impact of PPEs and other infection control measures that target all healthcare workers and not just high-risk workers. Implementation of such strategies will have challenges such as the lack of PPE availability, increased cost, and a reduction in the efficiency of care delivery. Additionally, our analysis shows that limiting the risk of hospital workers over 60 could result in a significant reduction in the overall morbidity and mortality amongst hospital workers [26, 29]. Currently, in the US, almost 30% of physicians and nurses are over the age of 60 years [30–32]. and removing such a large workforce from healthcare system will likely result in overall access to care. Innovative solutions, such as the use of telemedicine and reassignment to low-risk areas, could potentially be used to address the risk as well as access issues [24].

Our analysis has several limitations. We based our analysis on many assumptions that were true when we initially carried out the analysis. The current actual mortality in the US surpassed our estimates in the early days of the pandemic in the US. We believe the total number of healthcare worker infections and deaths are also likely greater than our estimates. Secondly, our analysis assumes that the level of exposure to US hospital workers is similar to those in Italy and China [21]. In the earlier phases of the pandemic, all three settings experienced a shortage of PPE [21, 25]. Thirdly, we assumed a similar population risk even though the overall population distribution and the average age of patients vary between the three settings [29, 33–35]. For the healthcare workforce, we controlled for the age distribution of the nurses and physicians in the US. We did not find data on other healthcare workers and assumed that their age distribution would be similar to nurses and physicians. Fourthly, the number of healthcare workers per bed is quite different in China, Italy and the US and was controlled in our analysis. We also assumed that the mortality rate amongst hospital will be three percent though the estimates for population level mortality have varied between 1.9 per million in South Korea to 258 per million to 395 per million in Lombardi, Italy. We were not able to do a sub-analysis of the mortality risk based on the presence of comorbidities like diabetes, heart diseases, and hypertension and did not include factors such as the shift length and number of hours working in the clinical areas [26]. Finally, we did not have the data to estimate the mental health morbidity due to exposure to COVID-19.

## Conclusion

We present a modeling study anchored in a particular time during the pandemic. Like all modeling studies, the conclusions are highly sensitive to the assumptions based on information available at the time of creating the model. Despite several months of the pandemic, the need to ensure availability and training on the use of PPEs across the healthcare system remains quite relevant in the US and internationally. Measures to reduce exposure of hospital workers above the age of 60 years could decrease the death rates among hospital workers by over 90%.

## Supporting information

S1 Fig Estimated number of COVID-19 related infections among healthcare workers in the United States based on Hubei and Italian scenarios. (TIF)
