# Supplementary figures and images for: Initial estimates of COVID-19 infections in hospital workers in the United States during the first wave of pandemic
Source: PLoS One. 2020 Dec 4;15(12):e0242589. doi: 10.1371/journal.pone.0242589 (PMC7717542; doi:10.1371/journal.pone.0242589)

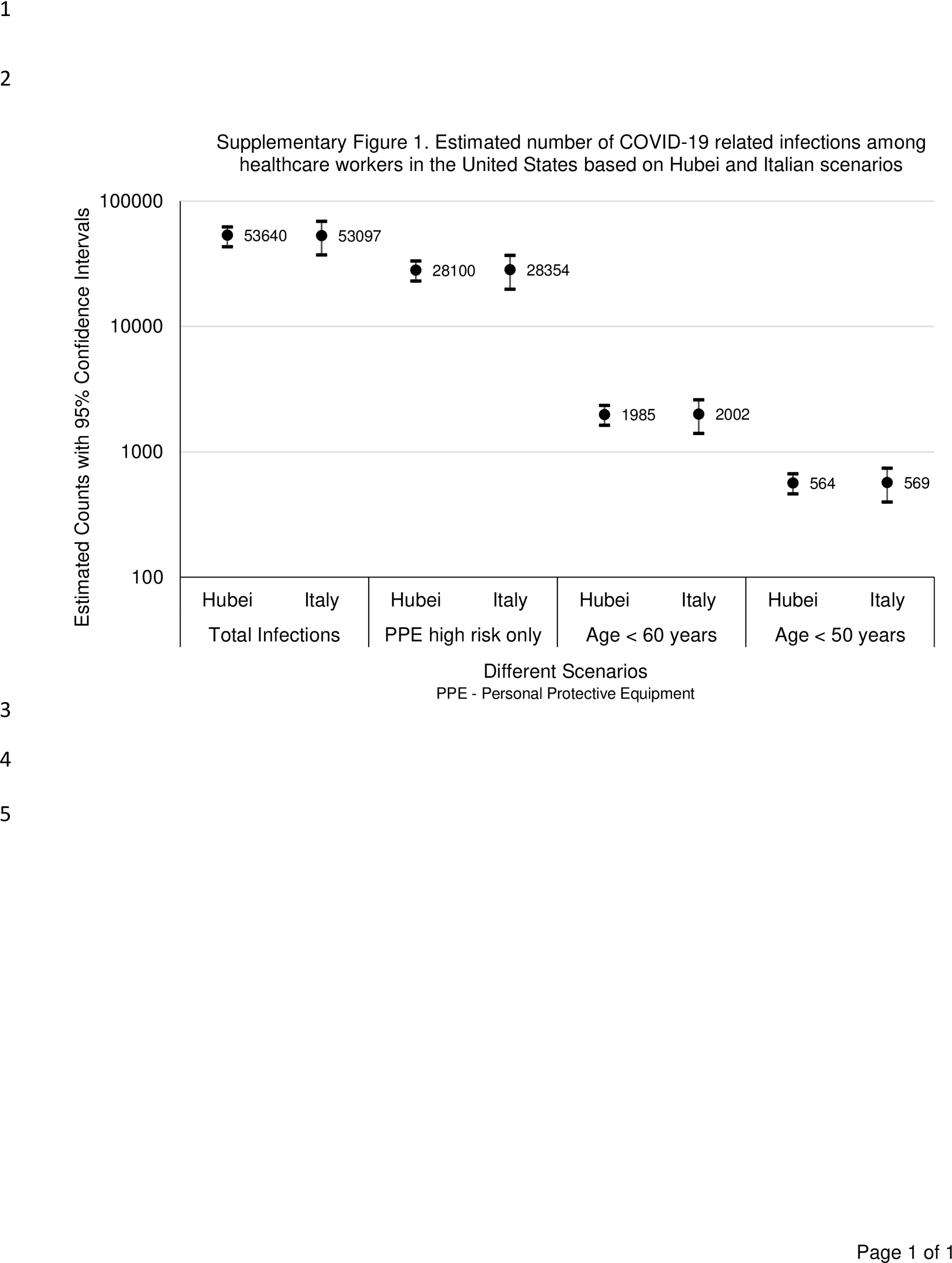

Supplement: S1 Fig — (TIF) [file pone.0242589.s001.tif]
